# Enabling stop codon read-through translation in bacteria as a probe for amyloid aggregation

**DOI:** 10.1038/s41598-017-12174-0

**Published:** 2017-09-19

**Authors:** Laura Molina-García, Rafael Giraldo

**Affiliations:** 1 Department of Cellular and Molecular Biology, Centro de Investigaciones Biológicas – CSIC, E28040 Madrid, Spain; 2 Present Address: Department of Cell and Developmental Biology, University College London, London, WC1E 6BT UK

## Abstract

Amyloid aggregation of the eukaryotic translation terminator eRF3/Sup35p, the [*PSI* ^+^] prion, empowers yeast ribosomes to read-through UGA stop codons. No similar functional prion, skipping a stop codon, has been found in *Escherichia coli*, a fact possibly due to the efficient back-up systems found in bacteria to rescue non-stop complexes. Here we report that engineering hydrophobic amyloidogenic repeats from a synthetic bacterial prion-like protein (RepA-WH1) into the *E. coli* releasing factor RF1 promotes its aggregation and enables ribosomes to continue with translation through a premature UAG stop codon located in a β-galactosidase reporter. To our knowledge, intended aggregation of a termination factor is a way to overcome the bacterial translation quality checkpoint that had not been reported so far. We also show the feasibility of using the amyloidogenic RF1 chimeras as a reliable, rapid and cost-effective system to screen for molecules inhibiting intracellular protein amyloidogenesis *in vivo*, by testing the effect on the chimeras of natural polyphenols with known anti-amyloidogenic properties. Resveratrol exhibits a clear amyloid-solubilizing effect in this assay, showing no toxicity to bacteria or interference with the enzymatic activity of β-galactosidase.

## Introduction

**Figure 1:**
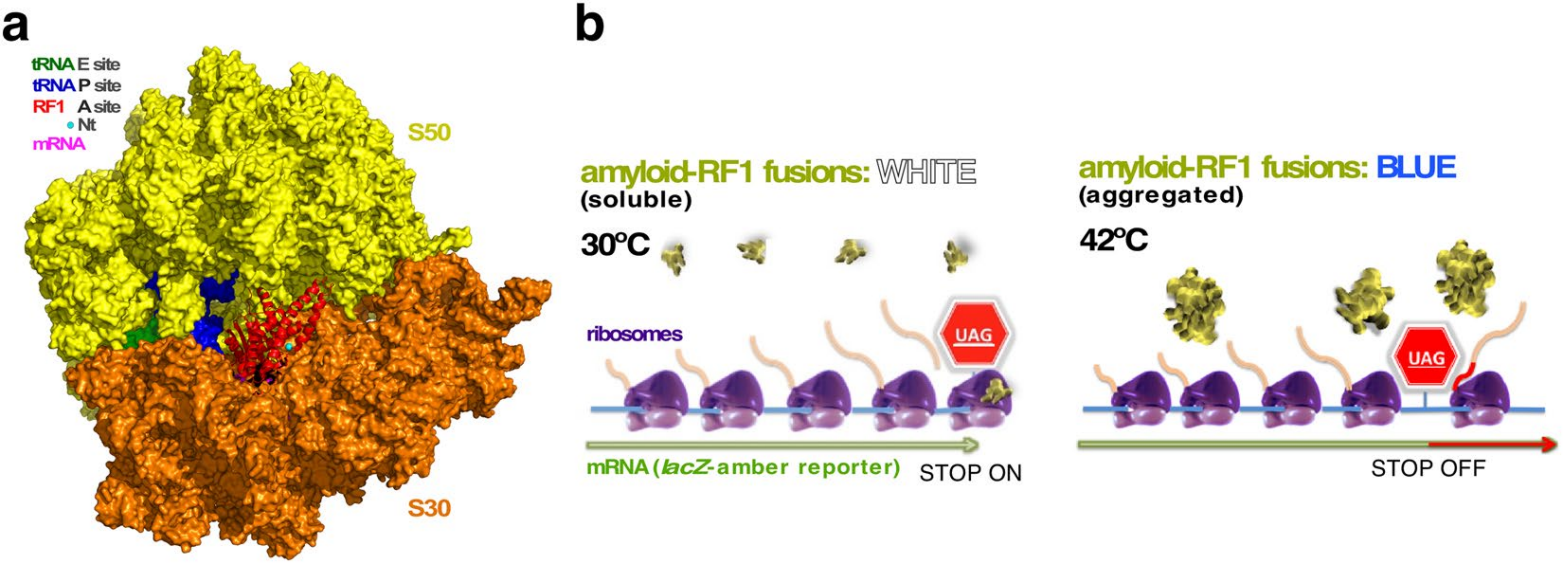
Amyloidogenic peptide tagging at the N-terminus of RF1 enables aggregation-dependent stop codons reading-through by *E. coli* ribosomes. (**a**) A bacterial ribosome assembled around a stop codon, showing the tRNAs bound at the P (peptidyl) and E (exit) sites and a RF1 molecule bound at the A (amino-acyl) site. The solvent-accessible N-terminus of RF1, where the WH1(Rn) peptides were attached is indicated. Structure rendered with PyMOL v.1.4 (https://www.pymol.org) on the PDB file 4v63. (**b**) A cartoon of the functional expansion in translation termination implemented in this report: bacterial ribosomes recognize or read-through amber stop codons depending on the solubility (*left*) or aggregation (*right*) of peptide-tagged RF1 (yellow).

The yeast prion [*PSI* ^+^], which is the amyloid-aggregated form of the Sup35p translation termination/ribosome releasing factor three (eRF3), expands the diversity of the proteome by reading through the UGA stop codon and is a widely studied basic model for amyloidogenesis and epigenetics^1^. In bacteria translation termination is dependent on the releasing factor 1 (RF1), which recognises the UAG and UAA stop codons, and releasing factor 2 (RF2) that recognizes UAA and UGA. Both factors are released from the ribosomal A site by the action of a GTPase (RF3, a Sup35p orthologue)^2,3^. The elucidation of bacterial ribosome structures in their different functional states has facilitated the characterization of the mechanism of action of RF1 and RF2^4,5^. These proteins are structural analogues of tRNAs that occupy the ribosomal A site over the termination codons, thereby placing amino acid side-chains to recognise the stop triplet, while projecting their respective N-terminal domains towards the exterior of the ribosome (Fig. 1a). Under conditions wherein the availability of RF1/RF2 is drastically reduced, when a stop codon is reached by the ribosome there is a pause in the translation and an anti-terminator aminoacylated tRNA inefficiently replaces RF1/2, thereby allowing for stop codon read-through in a discrete fraction of the pause events. The strict quality control exerted in *E. coli* ^6–8^, either on the stability of incompletely translated messengers or on partially synthesised proteins following a stop or a pause during elongation, may be a reason why translation termination in bacteria has not yet been exploited for biotechnological purposes, such as drug screening.

Model systems of protein amyloidoses, spanning from Alzheimer’s, Parkinson’s and prion neurodegenerative diseases to diabetes, have been successfully developed on the basis of eukaryotic organisms such as yeast, worms, flies, mice and, more recently, human stem cells^9^. Beyond their contribution to our understanding of amyloidoses, such models have enabled screening for anti-amyloidogenic compounds^10^. However, issues including the time needed to get a measurable signal or phenotype and, in the case of animal models, the requirement of fulfilling the two first of ‘the three Rs’ of Bioethics (the Replacement of animals and the Reduction in the number of them used)^11^, still press in favour of developing alternative screening platforms for biotechnological applications. Bacteria such as *Escherichia coli*, in spite of their ease and robustness of handling and the subsequent savings in cost and time, have been less used as proxies to amyloid diseases due to the evolutionary distance between prokaryotes and humans. There are a few exceptions, including extracellularly secreted bacterial amyloids, such as *curli*/CsgA^12^ and related amyloids, which scaffold biofilms and have been recently engineered to survey the amyloidogenic potential of any secreted protein and to test the anti-amyloidogenic activity of natural compounds^13^. More recently, bacteria have been also engineered to develop an assay in which the aggregation of β-lactamase in the periplasm, and thus the resistance levels against β-lactam antibiotics, is modulated by the aggregative potential of peptides fused to the enzyme^14^. Regarding intracellular amyloids, straight fusions of GFP to amyloidogenic peptides have been used to get a read-out of aggregation correlated with a loss in fluorescence emission^15^.

In this work, we have challenged the view that, upon ribosome stalling on a stop codon, the trans-translation checkpoint cannot be easily overcome in bacteria^6–8^, by exploring the feasibility of modifying one of the releasing factors in *E. coli* (RF1) to become inactivated through amyloid aggregation. Thus, we have generated chimeras between RF1 and a hydrophobic peptide of bacterial origin: the amyloidogenic stretch in the RepA-WH1 prionoid^16^. This prion-like protein was chosen as a proof of concept because it is the prototype of a bacterial intracellular amyloidosis, relevant as a model for human amyloid diseases since it elicits in *E. coli* a vertically (mother-to-daughter cells) transmissible amyloid proteinopathy^17^, propagating as alternative Hsp70 chaperone-modulated conformational strains^18^. Furthermore, RepA-WH1 exhibits cross-seeding both *in vitro* and *in vivo* ^17,19^, and shares pathways of toxicity with mitochondrial routes of amyloid disease (i.e., membrane targeting and ROS generation)^20,21^. The amyloidogenic, hydrophobic sequence in RepA-WH1 was recently found to functionally replace the polar repeated sequences that enable the yeast prion [*PSI* ^+^] to aggregate^22^. As a proof of concept, we also show that these chimeras constitute in bacteria a useful intracellular screening system for anti-amyloidogenic compounds, as described for analogous reporters built on [*PSI* ^+^] in yeast^23,24^.

## Results and Discussion

### Design of chimeras between amyloidogenic sequences and the *E. coli* releasing factor RF1

In yeast Sup35p, polar Q/N-rich sequences govern the aggregation of the protein as the [*PSI* ^+^] prion, which enables stop-codon read-through by ribosomes^1,23,24^. We have recently shown that 2-4 repeats of a hydrophobic and amyloidogenic sequence (LVLCAVSLI) from the bacterial prion-like protein RepA-WH1^25^, can functionally replace in yeast the polar Q/N oligopeptide repeats in [*PSI* ^+^] resulting in a new synthetic prion, [*REP-PSI* ^+^]^22^. To explore the possibility of generating an analogous synthetic prion in bacteria, thus enabling an epigenetic control on bacterial translation termination, we have now engineered the same amyloidogenic repeats of the RepA-WH1 sequence, in short WH1(R0-4), at the N-terminus of the *E. coli* RF1.

A strain carrying a thermosensitive allele of *prfA* ^26^, the gene encoding RF1, in which we had deleted the *lacZ* gene (MRA8-∆*lacZ*, *prfA* ^ts^) was used as host. In this strain, RF1^ts^ allows a normal termination of translation at UAG codons at 30 °C, whereas at 42 °C RF1^ts^ is disabled and thus translation termination is impaired, depending on complementation by a functional *prfA* gene. The expression of the WH1(R0-4)-RF1 chimeras in conditions promoting amyloidogenic aggregation (42 °C) would make translation termination defective, thus leading to read-through stop codons (Fig. 1b).

**Figure 2:**
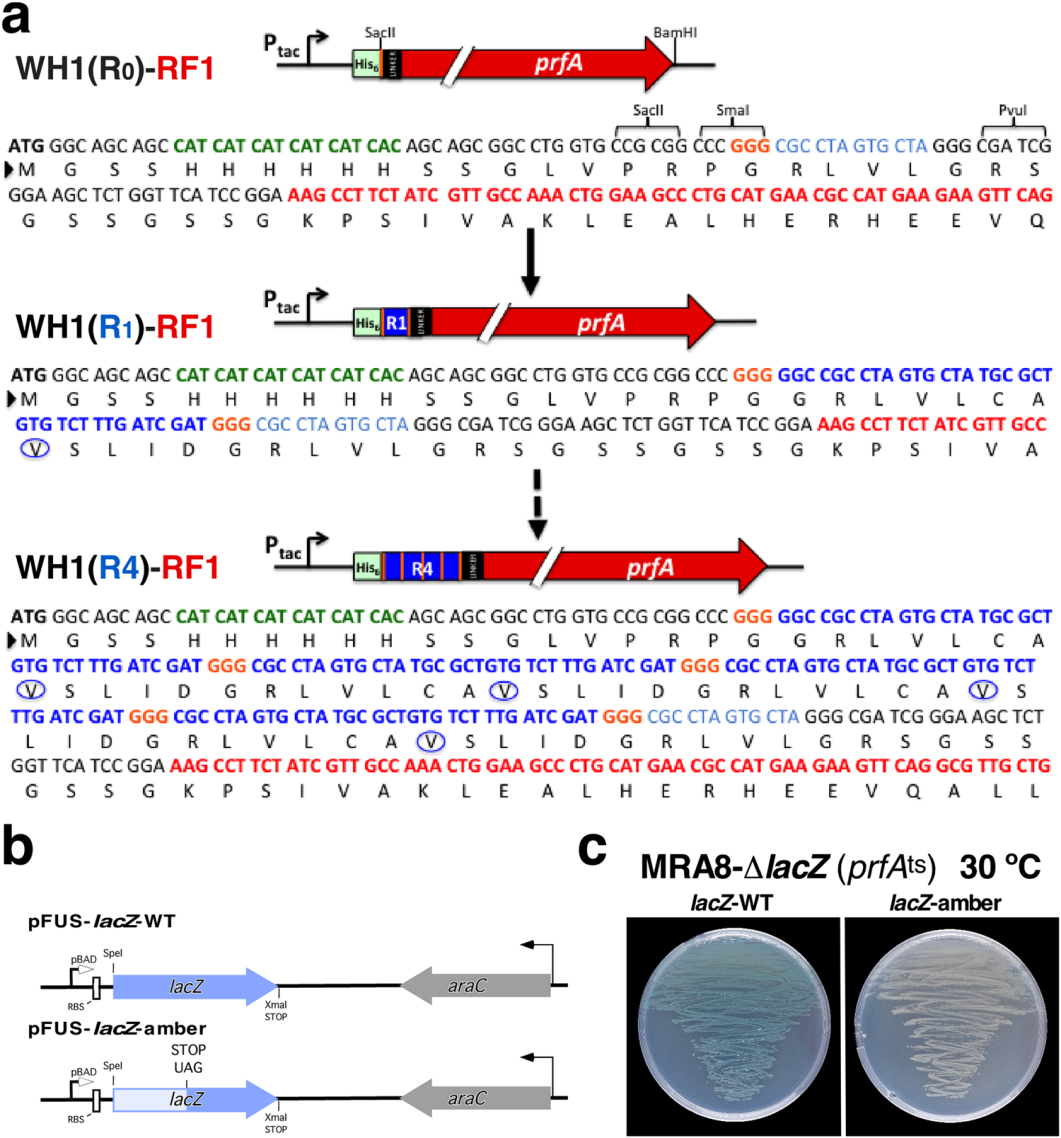
Design of the WH1(R0-4)-RF1 chimeras and the reporter *lacZ-WT/amber* plasmids. (**a**) The plasmid-borne fusions of the RepA-WH1 amyloidogenic peptides to *E. coli* RF1 (*prfA* gene). The control WH1(R0)-RF1 fusion (*top*) encodes at the N-terminus a His6-tag (green), two unique restriction sites and a flexible long linker (black) just before RF1 (red). The WH1(Rn)-RF1 chimeras accommodate the hydrophobic RepA-WH1 peptide repeats (blue; encircled: the hyper-amyloidogenic A31V mutation)^22^. Triplets encoding the Gly residues required to build the turn between the amyloidogenic repeats are also highlighted (orange). Just the single peptide repeat (R1, *middle*) and the final four repeats (R4, *bottom*) are displayed. (**b**) Arabinose-promoted expression (pBAD) cassette (*left*) with a wild-type *lacZ* reporter (blue) (*top*) or a mutated *lacZ-amber* variant carrying a premature UAG stop codon (*bottom*). (**c**) Functional assays in an *E. coli* MRA8*-∆lacZ*, *prfA* ^*ts*^ background. Complementation by the *lacZ-WT* construct leads to blue coloured bacteria (*left*). On the contrary, the expression of the *lacZ-amber* variant was unable to complement, due to the premature end of *lacZ-amber* translation (*right*). Experiment carried out at 30 °C because, in the absence of the WH1(Rn)-RF1 chimeras, translation termination is dependent on the host RF1^ts^.

A first plasmid construct series allowed for IPTG-induced expression of the His6-tagged WH1(R0-4) repeats fused to RF1, enabling complementation of the host RF1^ts^ (Fig. 2a). Two compatible reporter plasmids allowed for arabinose induction of *lacZ* by carrying either a UAA stop codon at its natural terminus (*lacZ-WT*), or an additional premature amber (UAG) mutation (*lacZ-amber*) generating a truncated and inactive enzyme (Fig. 2b). Such *lacZ-amber* reporter gives a blue or white coloration to bacteria depending, respectively, on the synthesis, or not, of the full length β-galactosidase enzyme (Fig. 2c).

### Aggregation of WH1(R2-4)-RF1 chimeras enables stop codon read-through in *E. coli*

**Figure 3:**
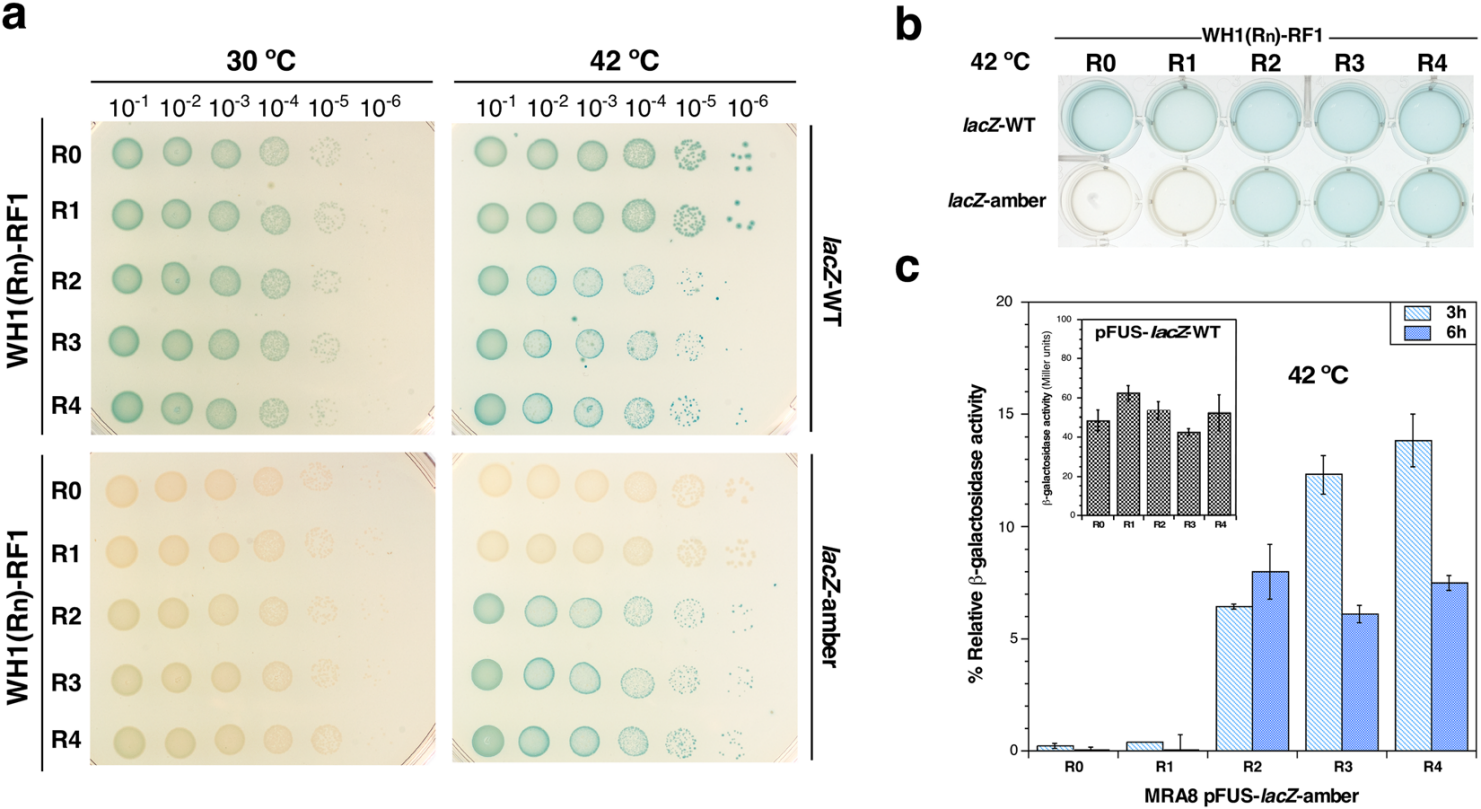
At least two WH1(Rn) repeats fused to RF1 are required to enable stop codon read-through. (**a**) Serial dilutions of bacterial cultures on LB-agar expressing the reporters *lacZ-WT* (*top*) or *lacZ-amber* (*bottom*), a variant carrying a premature stop codon, in the presence of a chromogenic substrate (X-Gal). Aggregation of the WH1(R2-4)-RF1 chimeras at 42 °C promotes amber codon read-through, while the chimeras that remain soluble, WH1(R0-1)-RF1, efficiently promote the end of translation. (**b**) A liquid culture assay in supplemented minimal medium. Again, blue colour (stop codon read-through) is observed for the WH1(R2-4)-RF1 chimeras in the *lacZ-amber* background. (**c**) β-galactosidase activity was determined in cells expressing the WH1(R0-4)-RF1 chimeras. Values show the percentage of activity for each chimera in combination with the *lacZ-amber* reporter, relative to the *lacZ-WT* reporter (inset). The histograms display the media and SDs (whiskers) from 4 independent replicas.

In cells expressing WH1(R0-4)-RF1 and carrying the *lacZ-WT* reporter, when grown at the permissive temperature (30 °C) on agar plates carrying the chromogenic substrate X-Gal, the characteristic blue-green colour was observed (Fig. 3a, *top-left*). In contrast, this blue-green colour was not observed in bacteria grown in the same conditions but carrying the *lacZ-amber* reporter, indicating an efficient translational termination, as expected (Fig. 3a, bottom-*left*). At the non-permissive temperature (42 °C), there was no apparent change, neither in colour nor in growth, in cells carrying the *lacZ-WT* reporter (Fig. 3a, *top-right*), due to the end of translation determined by RF2 at the terminal UAA stop codon. However, in the case of the *lacZ-amber* reporter only those bacteria bearing RF1 chimeras with at least two amyloidogenic repeats, WH1(R2-4)-RF1, displayed a blue-green colour at 42 °C (Fig. 3a, *bottom-right*). This pointed to the efficient read-through by the ribosomes of the premature amber codon in the high copy-number *lacZ-amber* mRNA, as a consequence of a loss of function in the WH1(R2-4)-RF1 chimeras. To test the ability of the engineered translation termination device to generate a signal amenable to high-throughput screening, bacteria were also grown in minimal liquid medium in multi-well plates. Colour phenotypes similar to those observed for the agar plates were obtained (Fig. 3b). Accordingly, β-galactosidase assays *in vitro* showed that at 42 °C the enzymatic activity for WH1(R2-4)-RF1 in *lacZ*-amber backgrounds, normalized to the corresponding value for the same chimeras in combination with the *lacZ*-WT reporter, increased progressively with the number of amyloidogenic tandem repeats, peaking at 3 h post-induction (Fig. 3c). With the *lacZ*-WT reporter, similar levels of β-galactosidase activity were achieved for all the chimeras, with no significant difference between the number of repeats in WH1(R2-4)-RF1 (Fig. 3c, *inset*).

Altogether, these results indicate that the aggregation of RFs can enhance stop codon read-through, and constitute a proof of concept on the feasibility of engineering a bacterial sensor for the amyloid aggregation of peptide stretches based on the same principle governing the [*PSI* ^+^] prion in yeast.

### Aggregation of WH1(R2-4)-RF1 chimeras is a suitable target for screening of anti-amyloidogenic compounds

**Figure 4:**
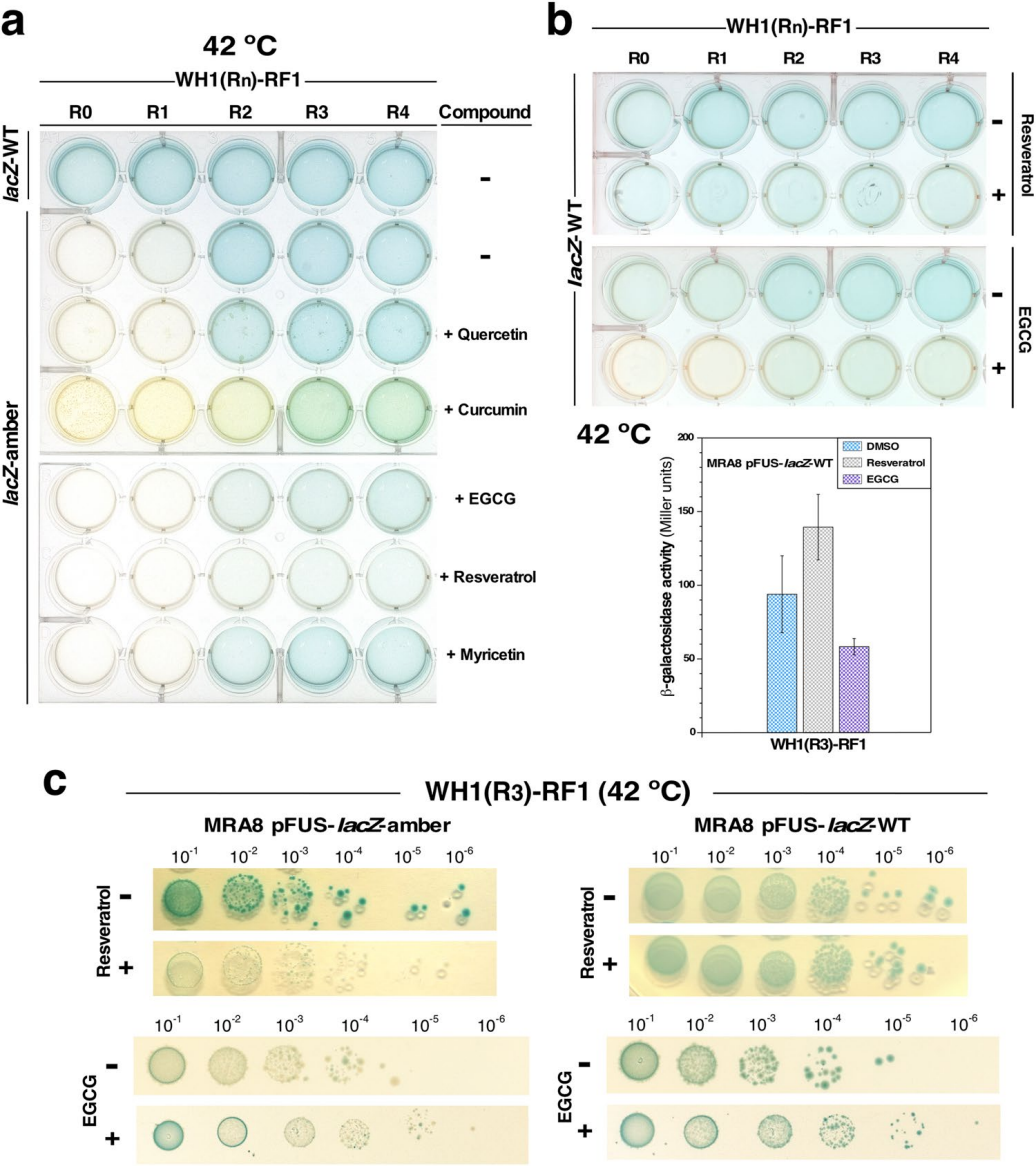
Screening the anti-amyloidogenic activity of polyphenolic compounds on WH1(R0-4)-RF1. (**a**) A multi-well plate assay (see Fig. 3b) in which the reading-through stop codon activity (blue colour in the *lacZ-amber* background) of the WH1(R0-4)-RF1 chimeras was tested in the presence of the indicated natural polyphenols. Resveratrol was the most efficient compound in reverting the read-through phenotype, i.e., lack of colour. (**b**) Resveratrol and EGCG (100 µM) were assayed in plates (*top*) and *in vitro* (*bottom*) in the *lacZ*-*WT* background, as a control for a possible effect of these compounds on β-galactosidase activity. EGCG, but not resveratrol, decreased the blue colouration (*top*), in a reaction not dependent on stop codon read-through. β-galactosidase assays (*bottom*) confirmed that, when compared with the co-solvent (DMSO), EGCG significantly decreased the activity of the enzyme, an evidence for its direct inhibition by this polyphenol. On the contrary, resveratrol apparently enhanced β-galactosidase activity. Data come from 6 independent replicas. (**c**) To further characterize the two more efficient compounds, their effects on the viability of bacteria expressing WH1(R3)-RF1 were checked through serial dilutions on agar plates. While resveratrol (*top*) did not show any significant effect on bacterial growth, colony size was consistently smaller upon EGCG treatment (*bottom*) in the *lacZ*-*amber* and *WT* backgrounds, an indication for some detrimental effect on bacterial physiology.

To determine the feasibility of using the bacterial sensor in screens for anti-amyloidogenic compounds, we tested a number of distinct polyphenolic molecules known to have such activity (Fig. 4)^10^. We found that, at 42 °C and with the *lacZ*-amber reporter, the sensor including the WH1(R2-4)-RF1 chimeras efficiently monitored the anti-amyloidogenic activity of resveratrol and, to a lesser extent, of epigallocathequin-3-gallate (EGCG) (Fig. 4a). These are two of the most active compounds in counteracting amyloidogenesis in a number of proteins involved in human disease^10^. An issue of concern in any sensor system is the possibility of having false positive results. To check if this were the case for WH1(R2-4)-RF1, the experiments with resveratrol and EGCG were repeated with the *lacZ-WT* reporter (Fig. 4b). Both the visual inspection of the liquid bacterial cultures (Fig. 4b, *top*) and β-galactosidase assays with the WH1(R3)-RF1 chimera (Fig. 4b, *bottom*) indicated that while resveratrol, compared to its carrier solvent DMSO, had some enhancing effect of the enzymatic activity of β-galactosidase, EGCG significantly inhibited the reporter. In addition, serial culture dilutions on agar plates showed that EGCG had some inhibitory effect on bacteria growth, because it led to a smaller size of colonies, while resveratrol did not have such effect (Fig. 4c). Therefore, the reporter system based on the WH1(R2-4)-RF1 and *lacZ*-amber is robust enough to screen for inhibitors of protein amyloidosis, provided that appropriate control assays on the compounds selected in the screening were performed. This must be also the case for any other reporter gene of choice (e.g., encoding luciferase or fluorescent proteins).

**Figure 5:**
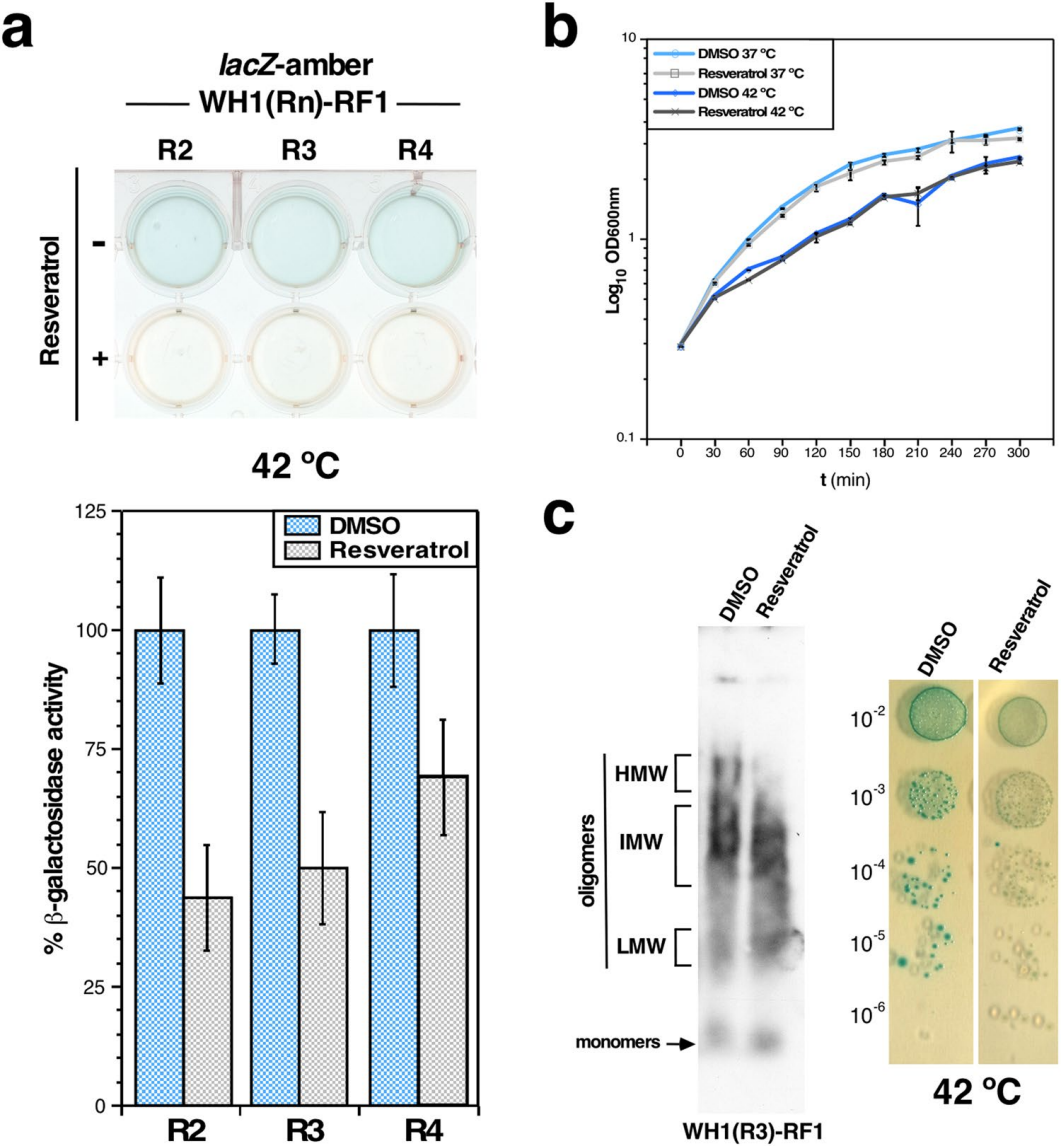
Resveratrol enhances solubility of the WH1(R2-4)-RF1 chimeras. (**a**) With a *lacZ-amber* reporter, resveratrol (100 µM) led to the loss of blue colour in the culture (*top*). β-galactosidase activity, normalized to that in DMSO, decreased in 40–60% for the chimeras (*bottom*; data from 6 independent assays). Both are indications of enhanced translation termination by ribosomes at the premature stop codon in the reporter. (**b**) At the concentration used in the assays (100 µM), resveratrol has no effect on the growth of *E. coli* cells at either of the temperatures used in the assays. (**c**) The effect of resveratrol on the aggregation of WH1(R3)-RF1 *in vivo* was assessed by SDD-AGE (*left*): the polyphenol (1 mM), but not its solvent (DMSO), reduced the population of high (HMW) molecular weight oligomers of the chimera, as expected from the solubilizing action of this polyphenol on amyloid aggregates^10^. As a control, 1 mM resveratrol does not have a detrimental effect on bacterial growth in the colour-based assay (*right*).

The effect of resveratrol on the WH1(R2-4)-RF1 chimeras was further characterized with the *lacZ*-amber reporter in liquid cultures *in vivo* (Fig. 5a). In β-galactosidase assays (Fig. 5a, *bottom*), as expected for a solubilizing effect of this polyphenol on the three chimeras, the levels of enzymatic activity, normalized to those measured for the untreated cells, decreased up to 50%, indicating a net increase in successful translation termination elicited by WH1(R2-4)-RF1. In parallel, we checked that resveratrol did not show any significant effect on the growth of bacteria (Fig. 5b).

To determine if the reduced stop codon read-through indeed was a consequence of decreased aggregation of the RF1 chimeras due to the action of the polyphenol, the effect of resveratrol on the WH1(R_3_)-RF1 chimera, which efficiently promoted amber stop codon read-through (Figs 3 and 4), was tested by semi-denaturing detergent agarose gel electrophoresis (SDD-AGE) (Fig. 5c). This is a technique capable to resolve large polydispersed amyloid aggregates on the basis of their resistance to solubilization by SDS^27^. Cell extracts from bacteria expressing WH1(R3)-RF1 in the presence of DMSO contained the chimeric protein as several oligomeric species, ranging from low to high molecular weights. However, those incubated in the presence of resveratrol showed a marked decrease in the amount of the largest aggregates. These results demonstrated that the reduction in the stop codon read-through by the ribosomes, as reported by the sensor, is a direct consequence of the ability of resveratrol to prevent the aggregation of the RF1 chimeras.

In parallel to the re-assignment of stop codons to code for non-natural amino acids^28–30^, this report shows that a translation termination factor (RF1) can also be engineered in bacteria to survey the propensity to amyloid aggregation of a given protein sequence. Moreover, we show here that amyloid-tagged RF1 is amenable for testing in bacteria compounds targeting intracellular amyloidosis. Future developments will imply to test chimeras of RF1 with peptide sequences involved in human amyloidoses and to explore the robustness of the assay in large-scale screenings. Once these issues have been addressed, the amyloidogenic RF1 chimeras, due to the handiness and simplicity of bacteria, may be included as a first *in vivo* screening assay in drug discovery programs targeting amyloid proteinopathies.

## Methods

### Construction of the WH1(R_0-4_)-RF1 chimeras

The *prfA* gene, encoding the bacterial translation termination factor RF1, was amplified by PCR from pELI02^26^. Besides a SacII site, which is necessary to clone the *prfA* gene, SmaI and PvuI targets were included at the 5′ end of the forward primer for the subsequent introduction of tandem repeats of the RepA-WH1(A31V) amyloidogenic peptide (L_26_VLCAVSLI_34_)^22,25^ (Fig. 2a). The amplified *prfA* was cloned into pRG-SD1, a vector carrying a suboptimal translation initiation sequence (to assure low intracellular levels of the cloned chimeras) and expressing hexahistidine fusion proteins under the control of an IPTG-inducible P_tac_ promoter^31^. The parental construction including *prfA* and none of the WH1 repeats was named pRG-WH1(R0)-RF1. Starting from this plasmid, the P_tac_-His_6_-WH1(R0)-RF1 module was transferred to a plasmid with a low copy-number RK2 replicon^18^, to get pRK2-WH1(R0)-RF1. For the construction of the rest of the chimeras, the inserts with the tandem repeats of the amyloidogenic peptide were obtained by means of enzymatic digestion (SmaI and EcoRV) of the pUKC1620_R1-4 plasmids^22^. Fragments were then ligated with pRG-WH1(R0)-RF1, previously digested with SmaI, to build pRG-WH1(R1-4)-RF1. Finally, the inserts containing the R1-4, repeats fused to *prfA* were transferred (SmaI and BamHI digestion) to pRK2-WH1(R0)-RF1, thus generating the pRK2-WH1(R1-4)-RF1 vector series.

### Construction of the pFus-*lacZ*-*WT/amber* vectors

The *lacZ* gene was amplified from pMLM132^32^ using oligonucleotides with SpeI (forward) and SmaI (reverse) 5′ ends. Both the PCR product and the pFus vector^33^ were digested with those enzymes and then fragments were ligated to give the pFus-*lacZ-WT* plasmid, which carried the *lacZ* gene under the control of the arabinose-inducible P_araBAD_ promoter. The pFus-*lacZ-amber* plasmid (Fig. 2b) was generated by means of site-directed mutagenesis using Pfu Turbo (Stratagene) and specific oligonucleotides to introduce a premature amber termination codon (UAG) at the position 1545–1547 of *lacZ* (LacZ residue change: A515*)^34^.

### Construction of the MRA8-*∆lacZ* strain

MRA8, an *E. coli* K-12 strain thermosensitive for RF1 due to the R137P mutation in the *prfA* gene^35^, was selected as host strain. The choice of a *prfA* ^ts^ rather than a null strain, was imposed by the fact that *prfA*/RF1 is essential in *E. coli* ^36^. MRA8 was transformed with pKD46, a plasmid expressing the λ-red recombinase under the control of the P_araBAD_ promoter^37^. Upon expression of λ-red (0.2% arabinose), 2 ml cultures were grown for 2 h at 30 °C and 1,100 rpm (Thermo Mixer Compact, Eppendorf). Cells were harvested and prepared in sterile H_2_O/glycerol for electroporation (MicroPulser, BioRad) with a PCR product including a *Km-parE* cassette flanked by 50 bp from the 5′ an 3′ ends of *lacZ*, to get the MRA8 *∆lacZ*::*Km-parE* strain. Using this strain as template, two 500-bp *lacZ* flanking regions were separately amplified by means of PCR. Both were ligated and then amplified, to generate a 1 kbp fragment that was used in a second recombination step in MRA8 *∆lacZ::Km-parE*, as described above. To select for the recombinant cells, platting was performed on M9 agar supplemented with 0.5% rhamnose, which induces the *parE* toxin^38^: only those cells having lost the *Km-parE* insert would grow under those conditions, giving place to the MRA8 *∆lacZ* strain. All constructs were sequenced (Secugen Inc., CIB-CSIC).

### Premature termination/read-through stop codon assays

All the assays were performed by expressing the WH1(R0-4)-RF1 chimeras in *E. coli* MRA8*-∆lacZ* cells, in such a way that genome-encoded RF1 is soluble and functional at 30 °C, but not at 42 °C. Cultures were grown in LB, or minimal medium plus casaminoacids (M9 + CAA), supplemented with 100 µg/ml ampicillin and 50 µg/ml kanamycin, overnight at 30 °C. On the following day, they were inoculated (1/100) into fresh medium and allowed to grow at 30 °C until they reached an OD_600nm_ of 0.3.

#### For the assays performed on agar plates

Serial dilutions of cultures were prepared (10^−1^–10^−5^). Subsequently, 7 µl drops of each dilution were added to LB plates with 100 µg/ml ampicillin, 50 µg/ml kanamycin, 0.02 mM IPTG, 0.001% arabinose, 0.003% glucose and 40 µg/ml X-Gal. Then bacteria were grown at 30 °C or 42 °C overnight to evaluate the read-through capacity of each chimera (i.e., colonies with a blue colouration).

#### For the assays performed in liquid

M9 + CAA, a colourless medium, was used. Five hundred µl of each culture (OD_600nm_ = 0.3) were added to a p24 multi-well plate (Falcon). Each well was supplemented with 0.02 mM IPTG, 0.001% arabinose, 0.003% glucose and 40 µg/ml X-Gal, and the plates were grown at 42 °C and 300 rpm (Thermo Mixer Compact, Eppendorf) for 24 h. The same protocol was used in the case of the assays with inhibitors of amyloid aggregation (Sigma; 25 mM stocks in DMSO): 50 µM curcumin, 100 µM quercetin, 100 µM epigallocatechin-3-gallate (EGCG), 100 µM resveratrol and 37.5 µM myricetin.

#### β-galactosidase assays

Cultures at OD_600nm_ = 0.3 were distributed into eight aliquots and the expression of β-galactosidase (LacZ) and the RF1 chimeras was simultaneously induced with 0.001% arabinose (plus 0.003% glucose) and 0.02 mM IPTG, respectively. Half of the samples were further grown at 30 °C and the other half at 42 °C. LacZ activity was evaluated in cell extracts by a colorimetric assay: the degradation of ONPG^39^. At least 3 independent experiments were performed for each chimera. For the assays performed in LB and with the *lacZ-WT* reporter, 200 µl culture aliquots were diluted to a final volume of 1 ml with Z buffer^39^, supplemented with 50 µl 0.1% SDS and 100 µl chloroform, and incubated at 28 °C for 10 min. Then ONPG (Sigma) was supplemented to 4 µg/ml and the reaction was left to proceed for 5 min, when it was stopped with 500 µl 1 M Na_2_CO_3_. For the chimeras combined with the *lacZ-amber* reporter, cells from 8 ml of culture were processed and the reaction was carried out for 15 min. In the case of the assays in M9 + CAA medium, 200 µl of cultures and a reaction time of 5 min were selected for *lacZ-WT*, whereas 300 µl and 15 min were used with the *lacZ-amber* reporter. With cells recovered from multi-well plates, the assay was performed at 24 h post-induction. β-galactosidase activity was quantitated according to the following formula^39^:

$${\rm{Miller}}\,{\rm{units}}=1000\,\times \,\{[{{\rm{A}}}_{420}-(1.75\,\times \,{{\rm{A}}}_{550})]/[{\rm{t}}({\rm{\min }})\,\times \,{\rm{V}}({\rm{ml}})\,\times \,{{\rm{OD}}}_{600}]\}$$

where A_420_ is the absorption of the ONPG degradation products; (1.75 × A_550_) a light scattering correction factor at 420 nm; and OD_600_, a correction for the number of cells.

### Testing the aggregation of the WH1(R3)-RF1 chimera: SDD-AGE

The presence of SDS-resistant amyloid oligomers, an indication for protein amyloidogenesis, was evaluated by means of semi-denaturing detergent agarose gel electrophoresis (SDD-AGE), as described^18,19^. Bacteria were grown at 30 °C to OD_600_ = 0.3, when they were supplemented with 1 mM IPTG and resveratrol (1 mM), or its solvent (DMSO) as a control, and then transferred to 42 °C for 3 h. The high concentrations of both the inducer and the polyphenol, compared with those used in the reporter β-galactosidase assays (see above), were due to the intrinsically limited sensitivity of SDD-AGE and, consequently, to the need of assuring sufficient intracellular amounts of the aggregates and their titration by resveratrol. Then harvested bacteria were mechanically lysed using a Fast-Prep (MP Biomedicals; 1.0 mm Ø Lysing Matrix C). Thirty µl of the bacterial cell lysates were resolved by electrophoresis in a 1.5% agarose-TAE gel supplemented with 0.1% SDS. Gels were then blotted to a PVDF membrane by wet electro-transfer (Mini Trans-blot, BioRad). Anti-His monoclonal (1/500) and anti-mouse polyclonal (1/5,000) antibodies (Sigma) were used in sequential incubations. Detection was performed with the ECL Plus luminescence kit (GE Healthcare) and AGFA Curix RP2 films.
